# Massively Enhanced Charge Selectivity, Ion Transport, and Osmotic Energy Conversion by Antiswelling Nanoconfined Hydrogels

**DOI:** 10.1021/acs.nanolett.4c03836

**Published:** 2024-09-05

**Authors:** Yi-Chuan Lin, Hong-Hsu Chen, Chien-Wei Chu, Li-Hsien Yeh

**Affiliations:** † Department of Chemical Engineering, National Taiwan University of Science and Technology, Taipei 10607, Taiwan; ‡ Department of Chemical Engineering, Feng Chia University, Taichung 40724, Taiwan; § Advanced Manufacturing Research Center, National Taiwan University of Science and Technology, Taipei 10607, Taiwan; ∥ Graduate Institute of Energy and Sustainability Technology, National Taiwan University of Science and Technology, Taipei 10607, Taiwan

**Keywords:** Nanofluidics, Nanoconfinement, Ion transport, Ion selectivity, Salinity gradient power

## Abstract

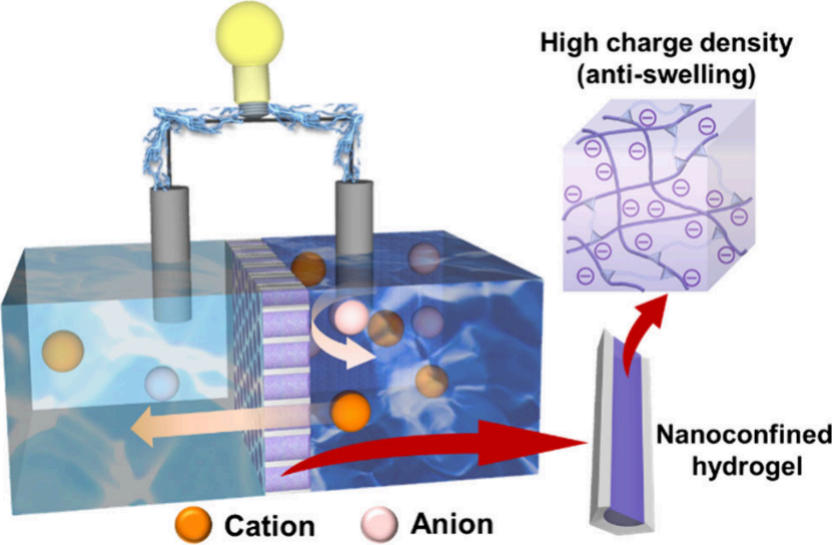

Developing a nanofluidic membrane with simultaneously enhanced ion selectivity and permeability for high-performance osmotic energy conversion has largely been unexplored. Here, we tackle this issue by the confinement of highly space-charged hydrogels within an orderedly aligned nanochannel array membrane. The nanoconfinement effect endows the hydrogel-based membrane with excellent antiswelling property. Furthermore, experimental and simulation results demonstrate that such a nanoconfined hydrogel membrane exhibits massively enhanced cation selectivity and ion transport properties. Consequently, an amazingly high power density up to ∼52.1 W/m^2^ with an unprecedented energy conversion efficiency of 37.5% can be reached by mixing simulated salt-lake water (5 M NaCl) and river water (0.01 M NaCl). Both efficiency indexes surpass those of most of the state-of-the-art nanofluidic membranes. This work offers insights into the design of highly ion-selective membranes to achieve ultrafast ion transport and high-performance osmotic energy harvesting.

Chemical potential energy stored in a concentration gradient, which is known as osmotic energy/power or salinity gradient power,^1−3^ can be directly transferred to electrical power via the reverse electrodialysis (RED) technique through ion-selective membranes.^4,5^ The RED-based osmotic energy has emerged as a promising sustainable energy source and attracted considerable attention in recent years, because it can harness natural osmotic gradients between freshwater and seawater interfaces for generating energy with an ultrahigh theoretical capacity up to ∼0.8 kWh m^–3^.^6,7^ To maximize practical osmotic energy output, various dimensional nanomaterials^8−22^ and fundamental approaches^23−26^ have been proposed and engineered for the application as nanofluidic ion-selective membranes in RED. For example, two-dimensional (2D) materials-based membranes with sub-2 nm channels^14−16^ or polyelectrolyte-like hydrogel membranes^17−20^ have been exploited for enhanced ion selectivity. The nanopore membrane with the directional ionic diode effect has been shown for reduced internal resistance and amplified ion permeability.^23−26^ However, an imbalance between ion selectivity and permeability of a nanofluidic membrane still affects the advancement of osmotic energy conversion efficiency.^5^ Until now, exploring a membrane featuring both enhanced charge selectivity and ion transport properties still remains considerably unexplored, which is highly necessary toward ultrahigh osmotic energy production.

In this work, we propose a physical approach to significantly resist the swelling of poly(2-acrylamido-2-methylpropanesulfonic acid) (PAMPS) hydrogel, achieved by confining it in the straight, high-porosity, and well-ordered channel arrays of alumina nanochannel membranes (ANMs) to form nanoconfined hydrogels (PAMPS@ANM). The orderly aligned channels of ANM make the PAMPS@ANM reduce resistance, and the nanoconfinement effect increases the space charge density of hydrogels. The membrane with confined hydrogels demonstrates a robust adhesion through the electrostatic interaction between the negatively charged PAMPS hydrogel plugs^19^ and the positively charged pore walls of ANM,^27^ endowing itself with excellent antiswelling property. Both experimental explorations and theoretical simulations were conducted to show the massively enhanced charge selectivity, ion transport, and thus osmotic energy conversion performance through the integration of space-charged hydrogels within well-aligned ANM channels. The optimization of osmotic power was also systematically investigated, considering various key parameters, such as channel size, ANM thickness, salt type, salinity gradient, and pH level. When the nanoconfined hydrogels were exposed to hypersaline conditions, such as the mixing of synthetic salt-lake water (5 M NaCl) and river water (0.01 M NaCl), an extraordinarily high power density up to ∼52.1 W/m^2^ can be achieved, significantly exceeding the typical values (ca. 10–30 W/m^2^) reported previously from most state-of-the-art nanofluidic membranes under the same operating conditions. This study offers important insights into the utilization of confined hydrogel materials for advanced osmotic energy harvesting technology.

All of the nanoconfined hydrogels (PAMPS@ANM) used were obtained by confining the highly space-charged PAMPS hydrogel into ordered nanopores of the ANM templates, which were constructed using the modified two-step anodization method^27,28^ (Figures 1a and S1; see details in the Supporting Information). Although the adopted inorganic ANM is brittle and lacks the toughness required for strong tensile performance, this work emphasizes their solid, hydroxyl-rich, highly ordered, and geometry-controllable nanoscale channels, which make them suitable candidates for investigating the nanoconfinement effect of antiswelling hydrogel and their potential to enhance ion transport and osmotic energy generation. Specifically, the H_2_O_2_-treated ANM was wetted with a mixed AMPS monomer solution, causing the infiltration of the AMPS monomer into ANM nanopores, leading to the formation of solution nanorods due to capillary force. Subsequently, the nanoconfined PAMPS hydrogels can be formed through a curing process involving UV irradiation, as verified by Fourier transform infrared (FTIR) spectra (Figure S2). The robust formation of the nanoconfined hydrogels is facilitated by the electrostatic interaction between negative charges carried by the PAMPS hydrogel and positively charged pore walls of ANM,^29^ enabling the confinement of the PAMPS hydrogel within the nanochannels. The scanning electron microscopy (SEM) images shown in Figure 1b,c prove the successful construction of the nanoconfined hydrogels in an ANM with highly aligned ∼75 nm channels, 36 μm thickness, and ∼8.3 × 10^9^ porse/cm^2^ pore density. Additional proof of the successful preparation of nanoconfined hydrogels is apparent through the decreased contact angle on the more hydrophilic surface of PAMPS@ANM (Figure 1d). The cross-sectional energy dispersive X-ray (EDX) mappings, which displayed a noticeable spatial sodium peak after 24 h immersion in a 5 M NaCl solution, provide the evidence of high cation selectivity of nanoconfined hydrogels (Figure 1e). The confinement of PAMPS hydrogels into highly aligned nanopores of ANM not only turns the ion-selective membrane from surface-charged to space-charged property but also provides low-resistance pathways for enhanced ion transport. As will be verified later, these features can contribute to enhanced ion selectivity and transport.

**Figure 1:**
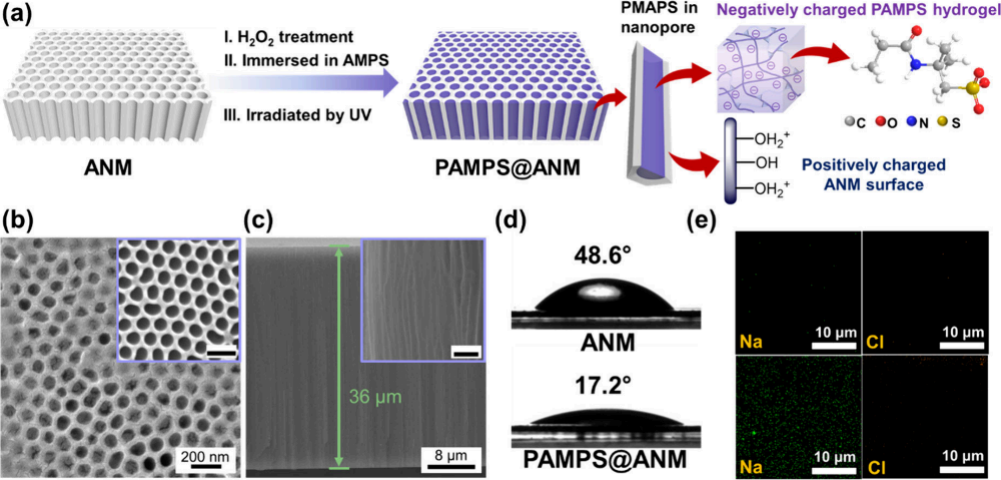
Construction and characterization of nanoconfined hydrogels (PAMPS@ANM). (a) Scheme describing the fabrication steps of PAMPS@ANM. The negatively charged PAMPS hydrogel was confined in the nanoporous ANM by incorporation of the interfacial interaction with positively charged walls of alumina nanochannels. (b) Top-view and (c) side-view SEM images of PAMPS@ANM, revealing the channels in ∼36-μm-thick ANM were plugged by the PAMPS hydrogel. Scale bars in insets of (b) and (c): 200 nm. Inset in (b) depicts the SEM image of the bare ANM, showing its pore size was ∼75 nm. Inset in (c) showcases the highly magnified image of nanoconfined hydrogels. (d) Contact angles of ANM and PAMPS@ANM. The decrease of contact angle of PAMPS@ANM implies the successful infiltration of PAMPS hydrogels in ANM. (e) EDX mappings of cross-sectional PAMPS@ANM before (upper panels) and after (bottom panels) immersion in a 5 M NaCl solution for 24 h. In the latter case, the much stronger sodium (Na) signal can be observed, demonstrating an excellent cation selectivity of PAMPS@ANM.

Typically, the hydrogel exhibits a severe swelling phenomenon in aqueous solutions, thus featuring the poor stability of network structures and low space charge density (upper panel in Figures 2a and S3). Such characteristics have discouraged the utilization of free hydrogels in harvesting osmotic energy. On the contrary, the nanoconfined hydrogel does not have these shortcomings. As shown in Figure 2a,b, the nanoconfinement effect significantly reduces the swelling degrees (*Q*_m_; see details in the Supporting Information) of nanoconfined hydrogels (PAMPS@ANM) under various NaCl concentrations (0, 0.01, 0.5, 1, and 5 M). For example, under the conditions of simulated river water (0.01 M NaCl) and seawater (0.5 M NaCl), the swelling degrees decreased approximately 94 and 91%, respectively, at conditions at which the PAMPS hydrogel is well confined in nanopores of an ANM (Table S1), indicating the excellent antiswelling efficacy of PAMPS@ANM. Note that slight swelling of the PAMPS hydrogel in the ANM still can be found, due to the fact that the hydrogel swelling may still occur along the open channel direction. Furthermore, the high space charge density caused by the nanoconfinement effect makes the PAMPS@ANM larger voltage-driven ionic conductance than the bare ANM in the low regime of salt concentrations, as verified by the stronger effect of the surface-charge-governed ion transport^30^ (i.e., larger difference in the ionic conductances between PAMPS@ANM and ANM at lower salt concentrations) shown in Figures 2c and S4. Under high salt concentrations, the conductance of PAMPS@ANM is slightly smaller than that of ANM. This can be attributed to the occupation of the nanochannels by the PAMPS hydrogel.

**Figure 2:**
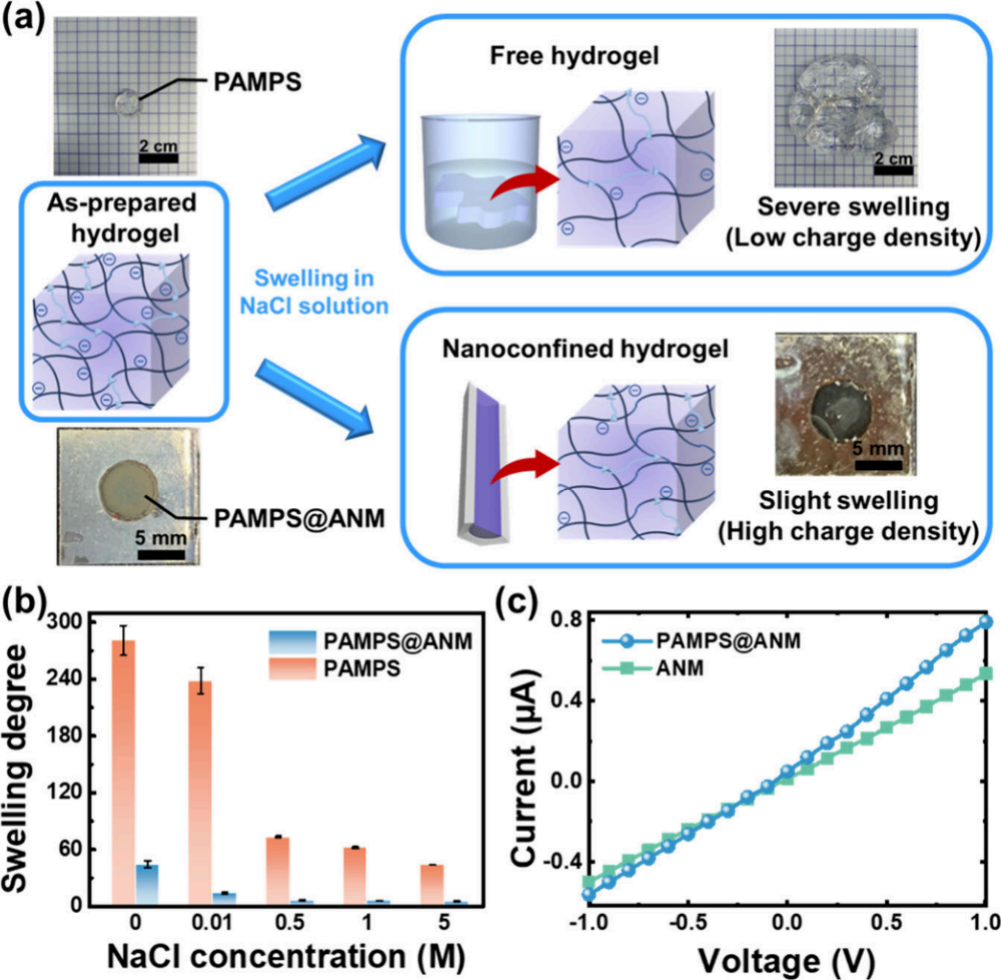
Antiswelling and enhanced ion transport behaviors. (a) Schemes and photographs depicting the swelling behaviors of free and confined PAMPS hydrogels in 0.01 M NaCl solution. The confinement effect gives the nanoconfined hydrogel much higher space charge density. (b) Equilibrium swelling degrees of free hydrogel (PAMPS) and nanoconfined hydrogels (PAMPS@ANM) in various NaCl concentrations. The apparent reduction of swelling degrees for PAMPS@ANM demonstrates the antiswelling strategy by physically trapping hydrogels in solid ANM nanopores. (c) Illustrated *I*–*V* curves of PAMPS@ANM and ANM, indicating the improvement of voltage-driven ion transport in the former.

As the next step, we explored the osmotic energy conversion efficiency of the developed nanoconfined hydrogels (Figure 3a). First, we compared osmotic ion transport behaviors of the ANM with and without confined PAMPS in a 0.5 M/0.01 M (50-fold) NaCl gradient. As shown in Figure 3b, the confinement effect significantly enhances the open-circuit voltage (*V*_oc_) and short-circuit current (*I*_sc_) by approximately 87.1 and 327%, respectively, due to the high space charge density of the PAMPS hydrogel in orderly confined pores of ANM. This suggests that the confined PAMPS hydrogel massively promotes ion selectivity and permeability of the modified membrane.^5^ Consequently, after calibration with the redox potential^31^ (Figure S5 and see details in the Supporting Information), it was estimated that the nanoconfined hydrogels can realize an ultrahigh energy conversion efficiency (η_max_) of 35.7% and a high cation selectivity (*t*^+^) of 0.922, both of which are also appreciably larger than those (η_max_, 2.23%; *t*^+^, 0.606) from the bare ANM (Table S2). Second, we compared the practical osmotic energy generation performances of the above two nanofluidic systems by transferring the generated current (*I*) to an external load resistor with adjustable resistance (*R*_L_), and thus the practical generated power under a salinity gradient can be calculated by *P* = *I*^2^ × *R*_L_.^27,32^Figure 3c reveals that the output current density of PAMPS@ANM is ∼3.3-fold larger than that of ANM, consistent with the finding of *I*_sc_ shown in Figure 3b. Benefiting from the massively enhanced ion selectivity as well as permeability by confined PAMPS hydrogels, Figure 3d shows that PAMPS@ANM can produce a maximum power density of ∼4.94 W/m^2^ (at *R*_L_ = 25 kΩ), which experiences a considerable ∼3.5-fold enhancement as compared to the bare ANM (∼1.10 W/m^2^ at *R*_L_ = 52 kΩ). All of the findings shown in Figures 2c and 3b–d and Table S2 clearly prove the main idea of this work that the confinement of highly space-charged PAMPS hydrogels in ordered nanochannels can effectively enhance the ion selectivity, ion transport, and osmotic energy performance of a hydrogel-modified membrane. Additionally, we conducted the testing area effect on the generated osmotic power density of PAMPS@ANM. As shown in Figure S6, the power density gradually decreases with the increase of testing area, which is expected due to the more significant effects of pore and pore interaction and ion concentration polarization (ICP).^33^

**Figure 3:**
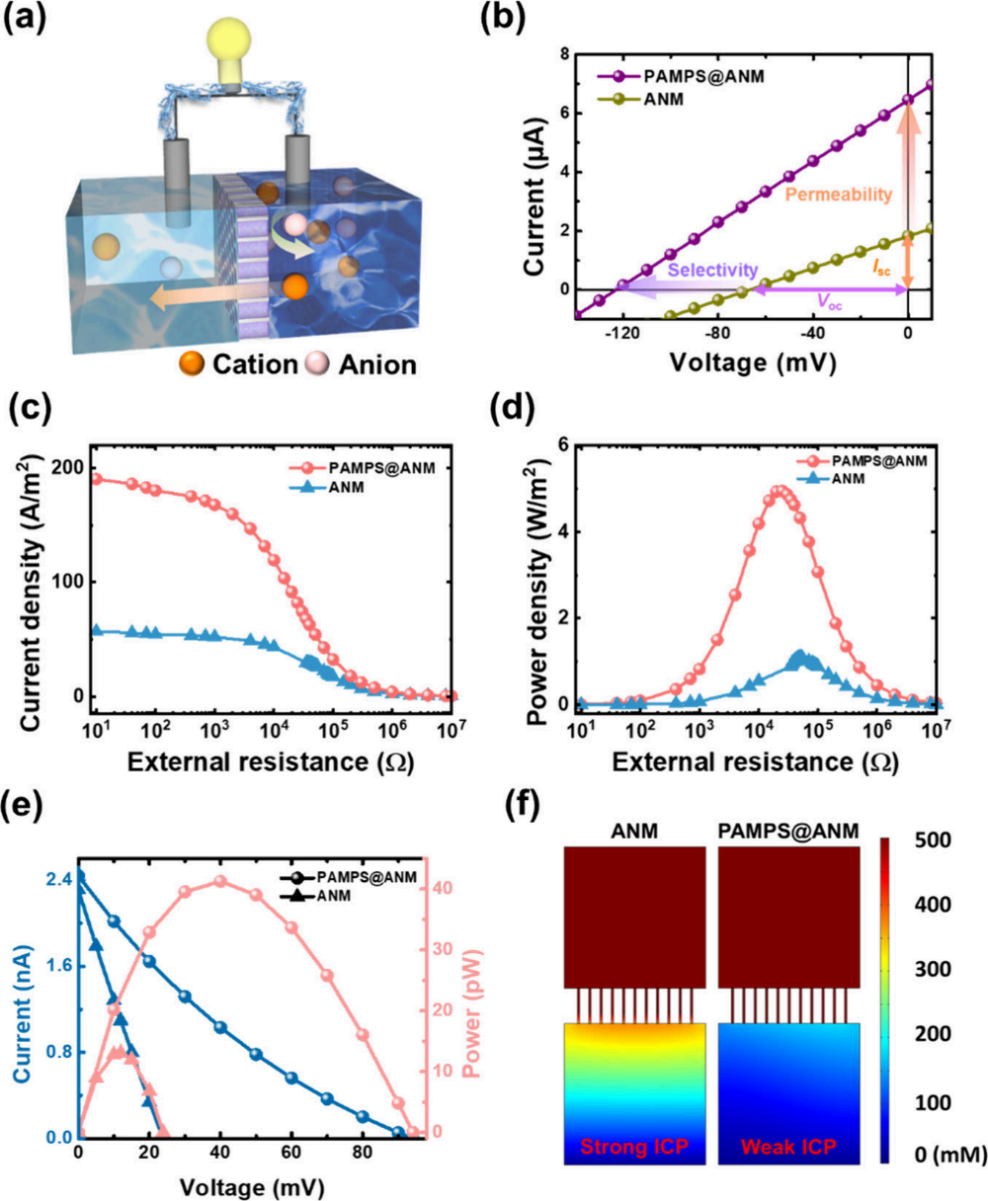
Confinement-induced enhancement of osmotic energy conversion. (a) Schematic of the experimental setup for directly harvesting energy from a concentration gradient using the cation-selective nanoconfined hydrogels. (b) Comparisons of *I*–*V* curves of PAMPS@ANM and ANM recorded under a 50-fold NaCl gradient. The produced *V*_oc_ and *I*_sc_ by ANM were 65.8 mV and 1.5 μA, respectively, and those by PAMPS@ANM were 123.1 mV and 6.4 μA. The confinement effect massively promotes the ion selectivity and permeability of nanoconfined hydrogels. Comparisons of output (c) current and (d) power densities of PAMPS@ANM and ANM as a function of external resistance under a 50-fold NaCl gradient. The maximum power densities produced by PAMPS@ANM and ANM were ca. 4.94 and 1.10 W/m^2^, respectively. (e) Simulated *I*–*V* and *P*–*V* curves and (f) spatial variations of total ionic concentration for the PAMPS@ANM and ANM systems in a 500 mM/10 mM NaCl gradient. The ICP effect is notably reduced for the PAMPS@ANM system.

To gain a thorough understanding of the mechanisms underlying the massively enhanced osmotic energy by nanoconfined hydrogels, we established the models to analyze the osmotic ion transport and energy conversion of the nanochannel array systems without (ANM) and with considering the polyelectrolyte-like PAMPS hydrogel (PAMPS@ANM) using the modified Poisson–Nernst–Planck and Stokes–Brinkman equations^25,34,35^ (Figure S7 and see detailed models in the Supporting Information). The simulations indicate that the confinement of the space-charged PAMPS hydrogels into the orderly straight nanochannel arrays is indeed able to increase the voltage, current, and power (Figure 3e), which agree consistently with the experimental findings shown above. Moreover, the PAMPS@ANM system shows less significant ICP effect^36−38^ than that from the ANM system (Figure 3f). Previous studies have shown that significant ICP effect would lead to the demotion of the effective concentration ratio within nanochannels, thus decreasing osmotic power conversion performance.^38,39^ Our model provides profound insights into why the nanoconfined hydrogels can massively improve osmotic energy conversion efficiency.

The design of the membrane plays a crucial role in attaining high-performance osmotic power generation. Consequently, parameter optimization emerges as a significant concern that demands careful consideration and a thorough investigation. To maximize the osmotic energy generation of the exploited nanoconfined hydrogels, various parameters including membrane thickness, ANM’s channel size, salinity gradient, salt type, and pH value have been explored. Notably, the benefits of employing ANM as a host membrane give the capacity to precisely regulate membrane thickness and channel size via well-controlled etching and electrochemical conditions throughout the two-step anodization processes (Figures 1b,c, S8, and S9; see details in the Supporting Information).^24,39^ The nanoconfined hydrogels show a local maximum trend with membrane thickness (Figure 4a); for example, the maximum power density increases from 3.89 W/m^2^ (21 μm) to 4.94 W/m^2^ (36 μm), and then decreases to 1.49 W/m^2^ (60 μm), as shown in Figure S10. The dependence is consistent with the channel-length-dependent osmotic power of the bare ANM^39^ and can be ascribed to the net consequence of the strong ICP effect at shorter thickness and the elevated resistance at greater thickness, both of which decline the osmotic energy performance.^37,39,40^ Shifting focus, we dedicated to the channel size effect by changing the ∼36 μm thickness membranes with varying channel sizes of ca. 40, 75, and 100 nm under a 50-fold NaCl gradient (Figures 4b and S11). As the channel size expands, the largely elevated ionic current and relatively consistent voltage lead to the decrease in the membrane resistance and thus a gradual uprising in osmotic energy. The maximum power density as high as ∼8.84 W/m^2^, significantly exceeding the industrial benchmark of 5 W/m^2^,^41^ at the internal resistance of 12 kΩ is reached when using the 100 nm pore diameter ANM as a template (Figure S11c). It is thus concluded that the channel size plays a key factor in promoting the osmotic energy conversion performance of the exploited nanoconfined hydrogels. Enlarging the channel size of the PAMPS@ANM can contribute to a considerably enhanced ionic flux while maintaining a sufficiently high level of ion selectivity, thus maximizing the osmotic energy output.

**Figure 4:**
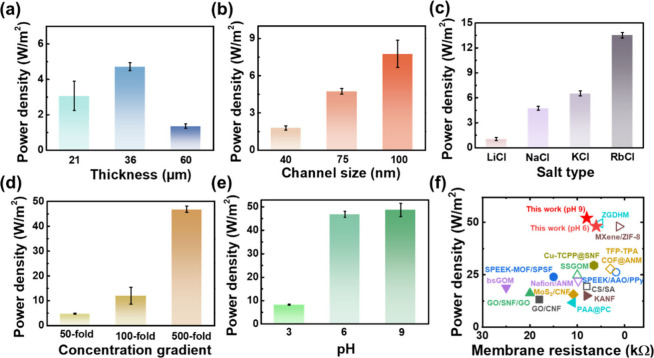
Optimization of osmotic energy generation. (a–e) Influences of the (a) membrane thickness, (b) channel size, (c) salt type, (d) NaCl concentration gradient, and (e) pH level on the osmotic power density produced by the nanoconfined hydrogels. (f) Comparison of the achieved maximum power densities and resistances among PAMPS@ANM and reported ion selective membranes under a 500-fold NaCl gradient. Closed symbols, membranes without an ionic diode effect; open symbols, membranes with an ionic diode effect.

As the final step, we investigated the effects of solution properties (salt type, concentration gradient, and pH) on the osmotic energy conversion performance of the exploited nanoconfined hydrogels (Figures 4c–e and S12–S15). The produced power densities under a fixed 50-fold concentration gradient of using different salts rank as RbCl (13.9 W/m^2^) > KCl (6.84 W/m^2^) > NaCl (4.94 W/m^2^) > LiCl (1.33W/m^2^), as shown in Figures S12 and S13 and Table S3. The observed trend closely mirrors the rise in diffusion coefficients of hydrated Li^+^ (1.03 × 10^–9^ m^2^ s^–1^), Na^+^ (1.33 × 10^–9^ m^2^ s^–1^), K^+^ (1.96 × 10^–9^ m^2^ s^–1^), and Rb^+^ (2.07 × 10^–9^ m^2^ s^–1^) ions, of which correspondence agrees with earlier findings.^42−44^Figure 4d further proves that the output osmotic power increases monotonically with the increase in the concentration gradient driving force. By mixing simulated salt-lake water (5 M NaCl) and river water (0.01 M NaCl), the nanoconfined hydrogels can reach a maximum power density of up to ∼48.2 W/m^2^ at the ultralow resistance of 6 kΩ, along with the *V*_oc_ of ∼202 mV and the ultrahigh energy conversion efficiency of 37.5% (Figure S14). Under such a hypersaline environment, the cation selectivity can still reach an ultrahigh value of 0.933. The nanoconfinement of the space-charged PAMPS hydrogels into highly ordered channel arrays of ANM massively enhances both ion selectivity and ion transport of the membrane, thus, lowering internal resistance and achieving ultrahigh-performance osmotic energy conversion. By elevating pH from 6 to 9, the maximum output power density can be further improved to 52.1 W/m^2^ (Figure S15), which largely surpasses the reported state-of-the-art ion selective membranes without ionic diode effect (closed symbols in Figure 4f and Table S4).^45−52^ Even comparing with the membranes with ionic diode effect (open symbols in Figure 4f and Table S4),^18,19,23,53−56^ which provides directional ion transport and thus lowers the membrane resistance, PAMPS@ANM also reaches the highest level. The near-saturation of osmotic energy harvesting performance from pH 6 to 9 can be attributed to the highly dissociable sulfonate functional groups of PAMPS, which could be nearly dissociated to negatively charged sulfonate anions (−SO_3_^–^) when pH ≥ 6.^19^

We also have demonstrated the practical applications of using the proposed nanoconfined hydrogels for charging commercial supercapacitors with various capacitances and powering a digital watch by connecting the osmotic energy generators in series (Figure S16). Figure S17 shows the high durability of the PAMPS@ANM, and the nanoconfined hydrogels can maintain the original porous structure after the durability tests, as verified in Figure S18.

In conclusion, we have experimentally and theoretically demonstrated the massive enhancements of ion selectivity, ion transport, and osmotic energy conversion efficiency by the confinement of highly space-charged PAMPS hydrogels into orderly aligned channel arrays of ANM templates. Moreover, the nanoconfinement effect makes the nanoconfined hydrogels have significantly enhanced antiswelling property. The optimization of the membrane design shows that an ultrahigh power density of 8.84 W/m^2^ can be produced by mixing simulated seawater (0.5 M NaCl) and river water (0.01 M NaCl). By elevating the NaCl gradient to 500-fold, the developed membrane can even generate the power density up to an unprecedented value of 52.1 W/m^2^ along with an ultrahigh energy conversion efficiency of 37.5% (corresponding an ultrahigh cation selectivity of 0.933), outperforming all existing ion selective membranes.^18,19,23,45−56^ This work presents a reliable strategy to overcome the trade-off between ion selectivity and permeability of nanofluidic membranes toward ultrahigh-performance osmotic energy conversion.

## Supporting Information Available

Experimental section, theoretical model, equilibrium swelling degree of the exploited nanoconfined hydrogels, additional experimental results, and comparison of the achieved power density from this work with reported state-of-the-art nanofluidic membranes (PDF)
