# Supplementary material for: Massively Enhanced Charge Selectivity, Ion Transport, and Osmotic Energy Conversion by Antiswelling Nanoconfined Hydrogels
Source: Nano Lett. 2024 Sep 5;24(37):11756–62. doi: 10.1021/acs.nanolett.4c03836 (PMC11421088; doi:10.1021/acs.nanolett.4c03836)
Supplement: Supplementary file 1 — nl4c03836_si_001.pdf [file nl4c03836_si_001.pdf]

Supporting Information

Massively Enhanced Charge Selectivity, Ion Transport and Osmotic Energy Conversion by Anti-Swelling Nanoconfined Hydrogels

Yi-Chuan Lin,[1] Hong-Hsu Chen,[2] Chien-Wei Chu,[1,2] and Li-Hsien Yeh[1,3,4*]*

[1]Department of Chemical Engineering, National Taiwan University of Science and Technology, Taipei 10607, Taiwan

[2]Department of Chemical Engineering, Feng Chia University, Taichung 40724, Taiwan

[3]Advanced Manufacturing Research Center, National Taiwan University of Science and Technology, Taipei 10607, Taiwan

[4]Graduate Institute of Energy and Sustainability Technology, National Taiwan University of Science and Technology, Taipei 10607, Taiwan

[*] Corresponding authors:

Email: lhyeh@mail.ntust.edu.tw (Li-Hsien Yeh)

cwchu@fcu.edu.tw (Chien-Wei Chu)

Experimental Section

Fabrication of ANM

All the ANM templates used were fabricated using the two-step anodization method, modified from our previous works.[1-2] In a typical procedure, a high-purity aluminum foil (99.9995%; thickness: 0.127 mm, Strem Chemicals) was electropolished using a perchloric acid/ethanol solution (1:4 v/v). The first anodization step involved using 0.3 M oxalic acid (Showa) electrolyte at 20 ℃ under a voltage of 50 V for 30 min. This was followed by a chemical etching process using a mixture of 6 wt% phosphoric acid (Honeywell) and 1.8 wt% chromium (VI) oxide (Showa) at 60 ℃ for 1 h. The second anodization step was then performed for 1, 2, or 4 h to regulate the membrane thicknesses[3] under the same conditions as the first anodization used. The channel sizes can be further adjusted by a channel-widening process using 5 wt% phosphoric acid at 30 ℃ for 30 or 50 min.[4]

Fabrication of PAMPS@ANM

The nanoconfined hydrogels (PAMPS@ANM) were constructed by infiltrating a mixed AMPS monomer solution into straight nanopores of an ANM, followed by a UV curing process. The mixed AMPS monomer solution consisted of AMPS (2.07 g, 10 mmol), *N*,*N'*-methylenebisacrylamide (MBAA, 7.7 mg, 0.05 mmol) as a cross-linker, 2-oxoglutaric acid (2-OA, 1.46 mg, 0.01 mmol) as photo-initiator, and 5 mL deionized (DI) water, where the used concentration of the AMPS monomer solution (C_{AMPS}) was calculated to be 29.2 wt%. To increase the wettability of the ANM surface with the mixed AMPS monomer solution, a pretreatment, aimed at enhancing the hydrophilicity and surface density of hydroxyl functional groups on the ANM channels [5], was carried out by immersing the ANM in a 30 wt% hydrogen peroxide (H_2O_2, Honeywell) solution for 30 min. The H_2O_2-treated ANM was then immersed in the AMPS solution for 2 min, after which any excess monomer solution on the outer surfaces of the ANM was carefully removed using Kimwipe tissues through capillary suction. To

fabricate the PAMPS@ANM, the AMPS solution within the nanochannels of ANM was cured by exposing it to a 365-nm UV irradiation for 30 min, achieved using a BK-Areacure-100100 LED module (Brightek) equipped with a planar UV light source at a power of 100 mW.

Material Characterizations

The morphologies of ANM and PAMPS@ANM were characterized using a JSM-7900F SEM (JEOL) at an accelerating voltage of 15 kV. For ion selectivity tests, the PAMPS@ANM was immersed in 5 M NaCl solution for 24 h and then rinsed with DI water. Afterward, the elemental analyses on the cross-section of the samples before and after NaCl immersion were conducted by the same SEM equipped with an EDX detector. Side-view images of water droplets on the ANM and PAMPS@ANM surfaces were captured using an OSA60-G contact angle instrument system (Ningbo NB Scientific Instruments) equipped with an optical surface analyzer. A 7 μL water droplet was used as the probing liquid for the measurements. The FTIR spectroscopy of PAMPS hydrogels was performed using a FT/IR-4X spectrometer (JASCO) employing the attenuated total reflection (ATR) method with a diamond crystal. The measurement was carried out in the spectral range of 650-4000 cm^{-1} with a resolution of 4 cm^{-1}.

Swelling Tests

The swelling tests were conducted by immersing PAMPS hydrogels or PAMPS@ANM in NaCl solutions with various concentrations (0, 0.01, 0.5, 1, and 5 M) at room temperature for different periods. The gel fraction (GF), defined by $m_{dry}/[m_{cured} \times C_{AMPS}]$, was measured to be 0.82, where m_{dry} and m_{cured} are the masses of dried hydrogel after swelling and as-cured hydrogel, respectively. The swelling degree (Q_m) in mass is defined as m_{gel}/m_{dry} and can be rewritten into $4.14\times(m_{gel}/m_{cured})$, considering the values of GF and C_{AMPS}. Specifically, for the PAMPS@ANM measurement, bare ANM (m_{ANM}), as-cured PAMPS@ANM ($m_{cured+ANM}$), and swollen PAMPS@ANM ($m_{gel+ANM}$) were recorded, sequentially. The Q_m for PAMPS@ANM

can be calculated based on the following equation:

$$Q_m = \frac{m_{gel}}{m_{dry}} = 4.14 \times \frac{m_{gel+ANM} - m_{ANM}}{m_{cured+ANM} - m_{ANM}} \quad \text{(S1)}$$

The equilibrium swelling degree is defined by the Q_m at equilibrium state after immersion in NaCl solutions for 1 day.

Electrical Measurement

The ion transport property and osmotic energy harvesting performance of the PAMPS@ANM were evaluated using a Keithley 6487 picoammeter (Keithley Instruments) connected to a custom-made conductive cell with a pair of Ag/AgCl electrodes. The membranes were positioned between two half-units of the conductive cell. To assess the voltage-driven ion transport property, current-voltage (*I–V*) curves were recorded in KCl with various concentrations. For the evaluation of the osmotic energy conversion performance, the working electrode was placed in the higher-concentration unit with various NaCl concentration gradients. The practical osmotic energy output can be estimated by transferring the produced power to an external resistor with a tunable resistance (R_t) and using the equation, $P = I^2 \times R_t$, where *I* is the measured current.[1] The effects of varying membrane thicknesses (~21, ~36, and ~60 μm) and channel sizes (~40, ~75, and ~100 nm) were investigated using the PAMPS@AMMs under a 50-fold NaCl concentration gradient while maintaining a constant low concentration of 0.01 M NaCl on one side. Furthermore, the impacts of different salt species (LiCl, NaCl, and KCl), salinity gradients (50-fold, 100-fold, 500-fold), and pH levels (3, 6, and 9) on the system were explored employing the PAMPS@ANM with a fixed channel size of 75 nm and membrane thickness of 36 μm. To ensure a fair comparison, all measurements were performed using an effective testing area of ~0.03 mm^2, as indicated in prior literatures.[6-8]

Electrode Calibration

In reference to the equivalent circuit diagram depicted in Figure S5a**,** the open-circuit voltage (V_{oc}) measured during the application of a salinity gradient across a selective membrane system can be decomposed into two distinct components: the osmotic potential (V_{osm}), and the redox potential (V_{red}) resulting from the non-uniform potential distribution at the two electrodes in the presence of a salinity gradient, which satisfy the following equation:

$$V_{oc} = V_{red} + V_{osm} \tag{S2}$$

To determine the pure salinity gradient-driven V_{osm} and osmotic current (I_{osm}), we initiated the electrode calibration using V_{red}. During this process, we recorded current–voltage curves while applying sweeping voltages ranging from −0.2 V to 0.2 V in increments of 0.01 V, both in the presence and absence of PAMPS@ANM. When PAMPS@ANM was absent, the measured voltage was designated as V_{red}. The recorded values of V_{red} were 46.7 and 78.3 mV in NaCl under the gradients of 50- and 500-fold, respectively.

Evaluation of Cation Selectivity and Maximum Energy Conversion Efficiency

The cation selectivity of the PAMPS@ANM can be determined by calculation of the cation transference number (t^+), which is obtained using the following equation:

$$t^+ = \frac{V_{osm}}{2\left(\frac{RT}{F}\right)\ln\left(\frac{\gamma_H C_H}{\gamma_L C_L}\right)} + 0.5 \tag{S3}$$

where γ represents the activity coefficients of salty solutions, and the subscripts H and L refer to the properties of salty solutions in high- and low-concentration reservoirs, respectively. In general, a t^+ value of 0.5 indicates a non-ion-selective membrane, while a t^+ value of 1.0 signifies an ideally cation-selective membrane.

Upon obtaining t^+, we can determine the maximum osmotic energy conversion efficiency (η_{max}) of the PAMPS@ANM using the following equation:

$$\eta_{max} = \frac{\left(2t^+ - 1\right)^2}{2} \times 100\% \tag{S4}$$

Theoretical Model

To reduce computational cost, we simplified the PAMPS hydrogel plugs in ANM (surface charge: +0.08 C/m^2)[9] as dense polyelectrolyte layers with uniformly high space charge density of -1.7×10^8 C/m^3. Figure S7 depicts the simulated PAMPS@ANM system under consideration, and the ANM system is similar to Figure S7 in the absence of polyelectrolyte hydrogel plugs. Osmotic ion transport and energy conversion of the above systems can be described by the coupled Poisson-Nernst-Planck and Stokes-Brinkman equations,[10-12]

$$-\nabla^2 V = \frac{\sum_{i=1}^{2} F z_i C_i + \Phi \rho_{gel}}{\varepsilon} \tag{S5}$$

$$\nabla \cdot \mathbf{J}_i = \nabla \cdot \left(\mathbf{u} C_i - D_i \nabla C_i - \frac{F z_i C_i D_i}{RT} \nabla V \right) = 0,\ i = 1, 2 \tag{S6}$$

$$\mu \nabla^2 \mathbf{u} - \nabla p - \nabla V \left(\sum_{i=1}^{2} F z_i C_i \right) - \Phi \frac{\mu}{\left(\lambda_{gel} \right)^2} \mathbf{u} = \mathbf{0} \tag{S7}$$

$$\nabla \cdot \mathbf{u} = 0 \tag{S8}$$

In the above, ρ_{gel} is the space charge density of the PAMPS hydrogel; V, F, and R are the electrical potential, Faraday constant, and gas constant, respectively; ε, T, $\mathbf{u}$, p, and μ are the permittivity, temperature, velocity, pressure, and dynamic viscosity of fluid, respectively; z_i, C_i, $\mathbf{J}_\mathbf{i}$, and D_i are the valence, concentration, flux, and diffusivity of ith ionic species ($i = 1$ for cations and $i = 2$ for anions); Φ is the region function ($\Phi = 1$ represents the region inside the hydrogel and $\Phi = 0$ represents the region outside the hydrogel); λ_{gel} is the softness degree of hydrogel. For the bare ANM system, we assumed $\rho_{gel} = \lambda_{gel} = 0$.

The ionic current through the simulation system can be calculated by using

$$I = \int_S \left(\sum_{i=1}^{2} F z_i \mathbf{J}_i \right) \cdot \mathbf{n} dS, \tag{S9}$$

where *S* denotes either end of the two reservoirs. The total meshes used in the modeling are

about 500,000.

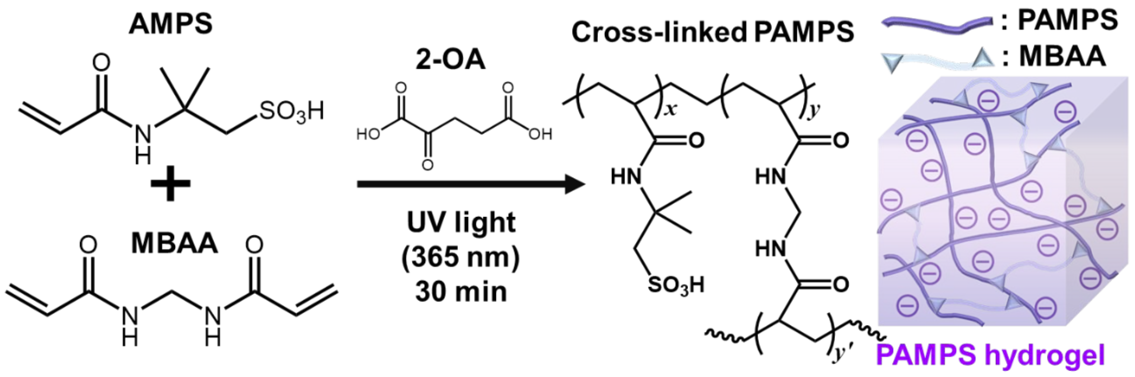

Figure S1. Synthesis pathway of the PAMPS hydrogel through UV curing.

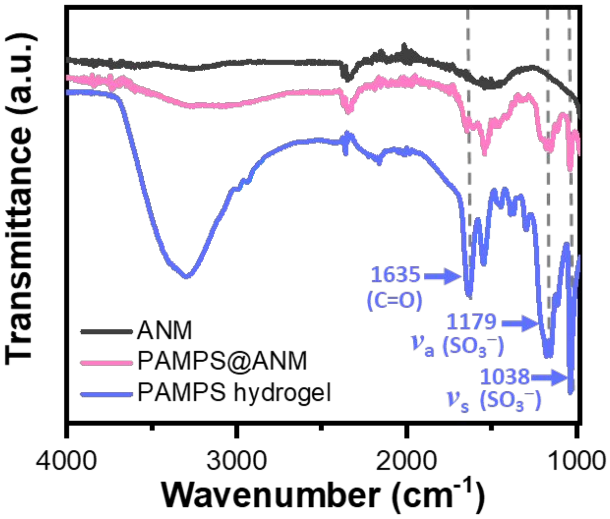

Figure S2. FTIR spectra of the pure PAMPS hydrogel, ANM and PAMPS@ANM. The peak observed at 1635 cm^{-1} corresponds to the stretching of C=O in amide linkage, while those observed at 1179 and 1038 cm^{-1} correspond to the asymmetric and symmetric stretching of SO_3^-, respectively, indicating successful preparation of the nanoconfined PAMPS hydrogels.

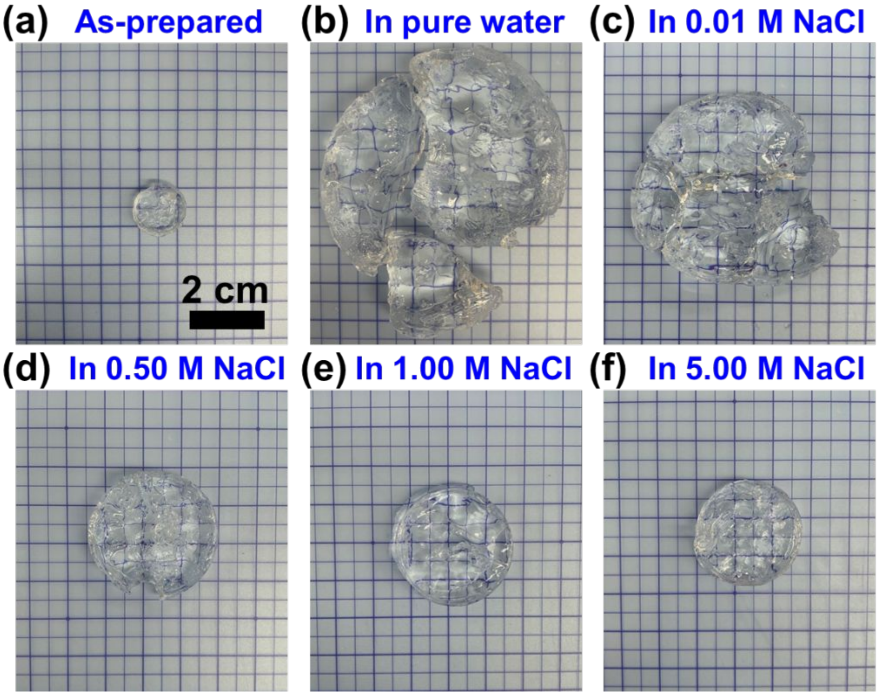

Figure S3. Photos of PAMPS hydrogels at different states: (a) as-prepared and (b-f) after swelling in (b) pure water, (c) 0.01 M NaCl, (d) 0.50 M NaCl, (e) 1.00 M NaCl, and (f) 5.00 M NaCl. Significant swelling of the PAMPS hydrogel was observed in unconfined environments and the swelling became much more severe at lower salt concentrations, resulting in more deformed and broken network structures.

Table S1. Equilibrium swelling degrees of PAMPS and PAMPS@ANM in NaCl solutions with various concentrations.

**NaCl Concentration (M)**	**Swelling degree (g/g)**	
	**PAMPS (Free hydrogel)**	**PAMPS@ANM (Nanoconfined hydrogel)**
0.00	280.7 ± 15.7	44.5 ± 3.6
0.01	238.2 ± 13.6	14.2 ± 0.9
0.50	73.4 ± 0.8	6.5 ± 0.4
1.00	65.3 ± 0.8	5.9 ± 0.3
5.00	43.8 ± 0.3	5.6 ± 0.2

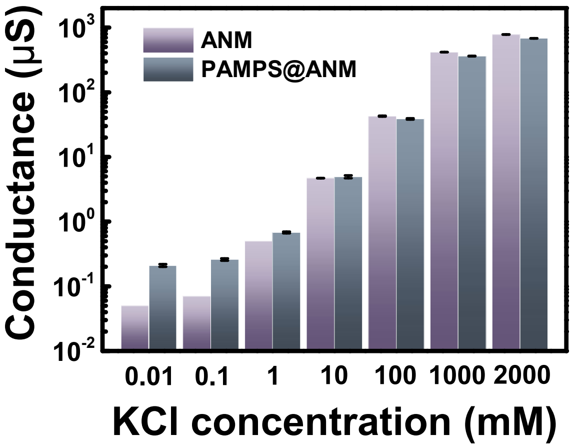

Figure S4. Comparison of ionic conductances of PAMPS@ANM and ANM under various KCl concentrations.

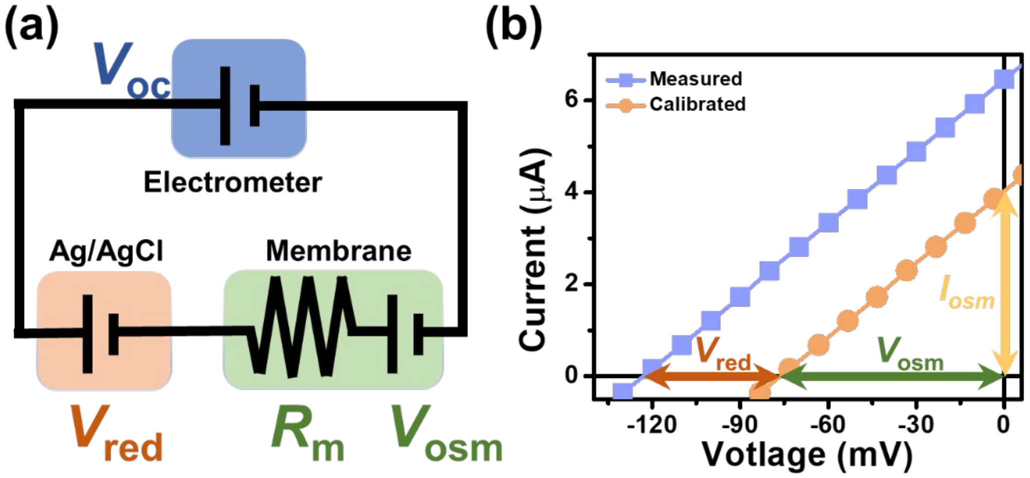

Figure S5. (a) Scheme depicting the equivalent circuit diagram of the considered osmotic energy conversion system. The measured open-circuit voltage (V_{oc}) is composed of two components: the redox potential (V_{red}) at the Ag/AgCl electrodes and the pure salinity gradient-driven osmotic potential (V_{osm}). Only the V_{osm} contributes from the ion-selective membrane subjected to a salinity gradient. R_m represents the internal resistance of the membrane. (b) The illustrative I–V curves of PAMPS@ANM under a 50-fold NaCl gradient (0.5 M/0.01 M) before (squares with a line) and after (spheres with a line) the redox potential calibration process. The values of V_{osm} and I_{osm} can be directly determined from the intercepts on the voltage and current axes of the calibrated curve, respectively.

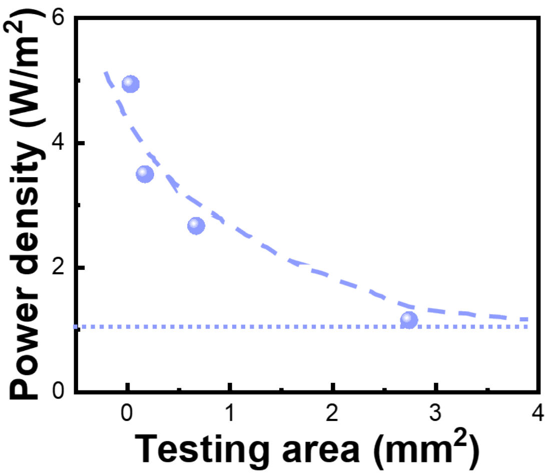

Figure S6. Effect of the testing area on the generated power density of PAMPS@ANM. The applied salinity gradient is 50-fold NaCl concentration gradient. As the testing area increases, the osmotic power density gradually decreases and approaches an asymptotic value of ~1.15 W/m^2.

Table S2. Osmotic energy conversion efficiencies of the ANM without and with confined PAMPS tested under a 50-fold NaCl gradient.

Efficiency	0.5 M/ 0.01 M NaCl	
	**ANM**	**PAMPS@ANM**
V_{oc} (mV)	65.8	123.1
V_{red} (mV)	46.7	46.7
V_{osm} (mV)	19.1	76.4
I_{sc} (μA)	1.5	6.4
I_{osm} (μA)	0.4	4.0
t^{+}	0.606	0.922
η_{max} (%)	2.23	35.7
Internal resistance (kΩ)	52	25
Maximum power density (W/m^2)	1.10	4.94

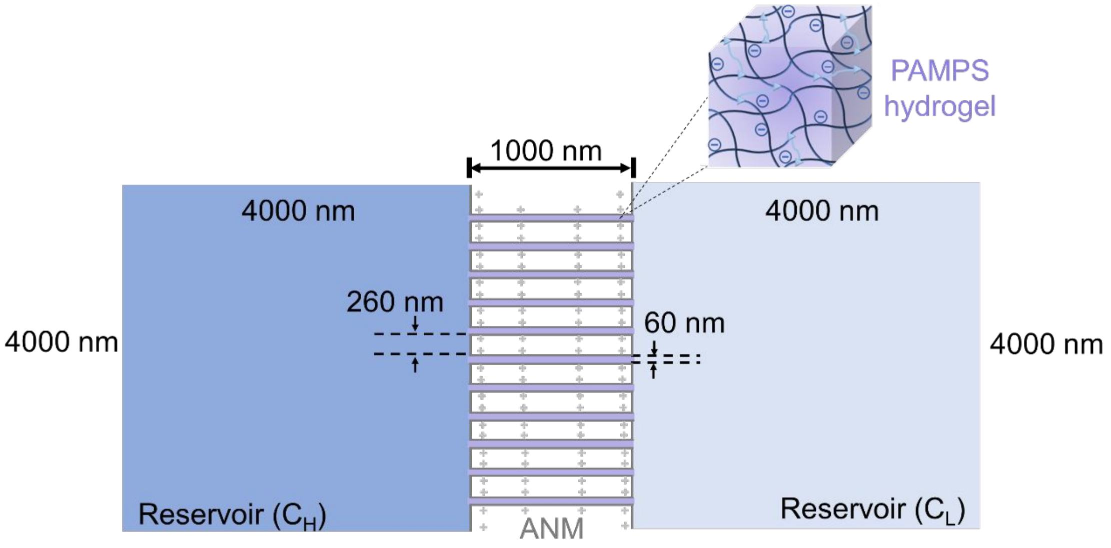

Figure S7. Schematic representation of the simulated PAMPS@ANM system (not to actual scale). The PAMPS hydrogel is conceptualized as a negatively charged polyelectrolyte plug carrying a space charge density of -1.7×10^8 C/m^3, located within 11 channel arrays of ANM bearing a surface charge density of +0.08 C/m^2. For the bare ANM system, the polyelectrolyte plugs were omitted.

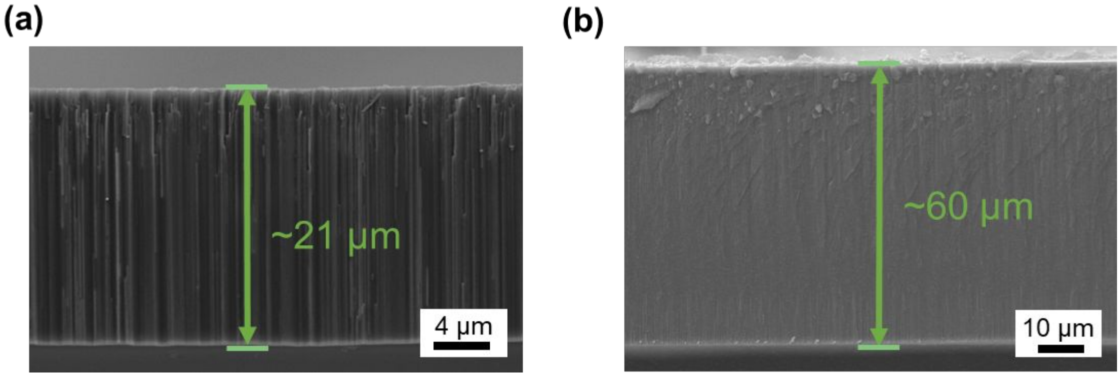

Figure S8. SEM images of PAMPS@ANM with thicknesses of (a) ~21 and (b) ~60 μm.

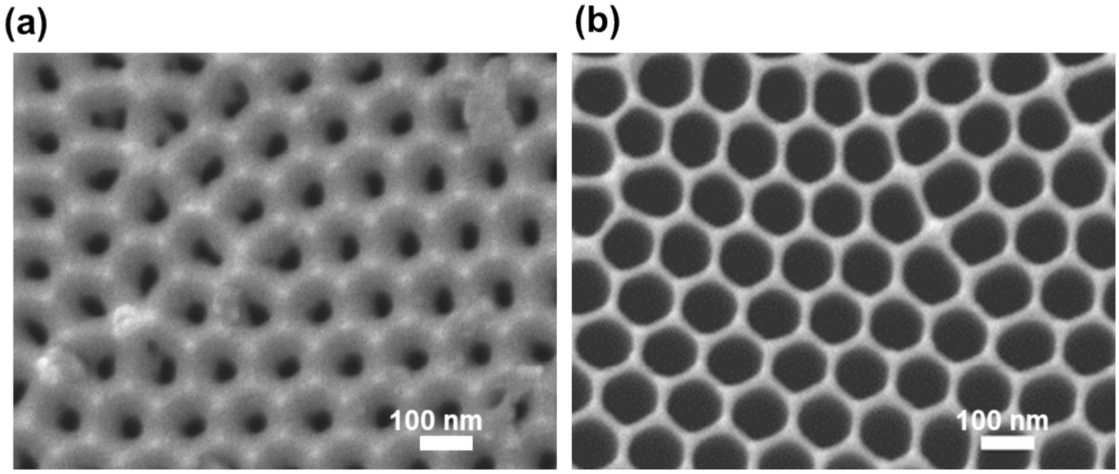

Figure S9. SEM images of ANM templates with channel sizes of (a) ~40 and (b) ~100 nm.

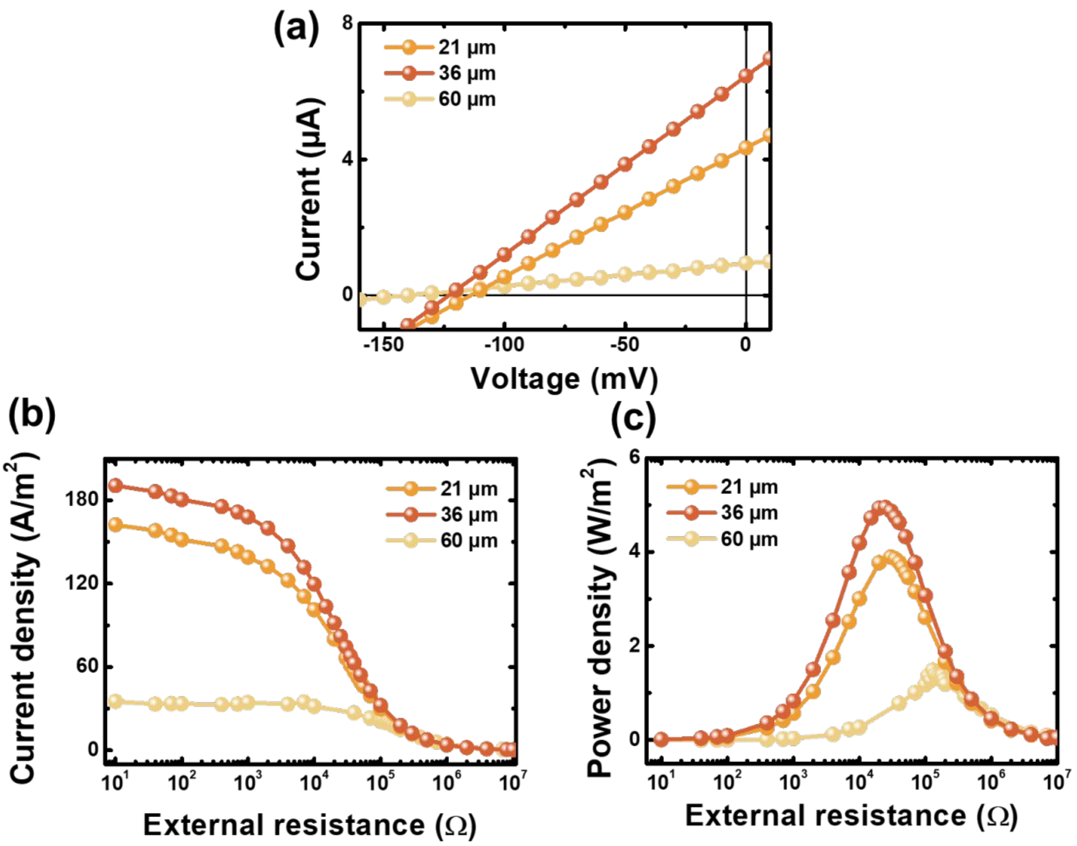

Figure S10. Illustrated (a) *I*-*V* responses, (b) current densities, and (c) power densities of PAMPS@ANM with different thicknesses under a 50-fold NaCl gradient. The maximum power densities achieved by 21, 36, and 60 μm in thickness membranes were 3.89, 4.94, and 1.49 W/m^2, respectively.

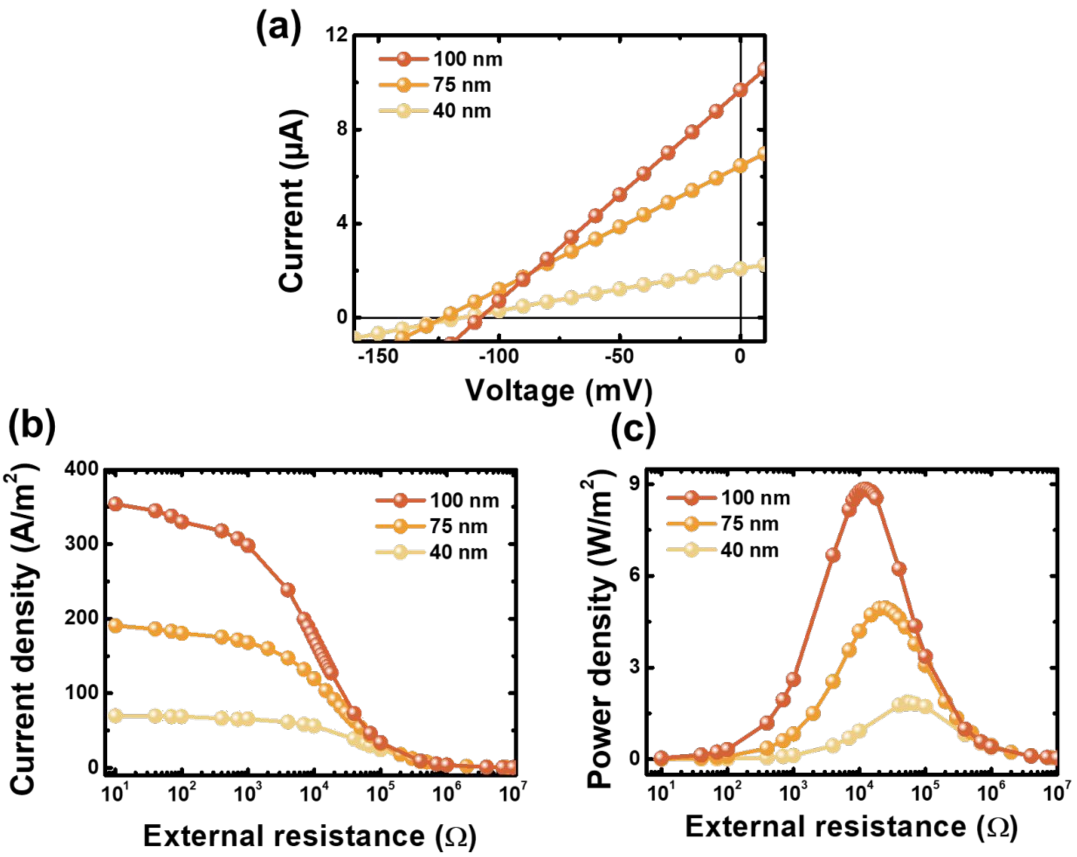

Figure S11. Illustrated (a) *I-V* responses, (b) current densities, and (c) power densities of PAMPS@ANM with different channel sizes under a 50-fold NaCl gradient. The maximum power densities achieved by 40, 75, and 100 nm in pore diameter membranes were 1.86, 4.94, and 8.84 W/m^2, respectively.

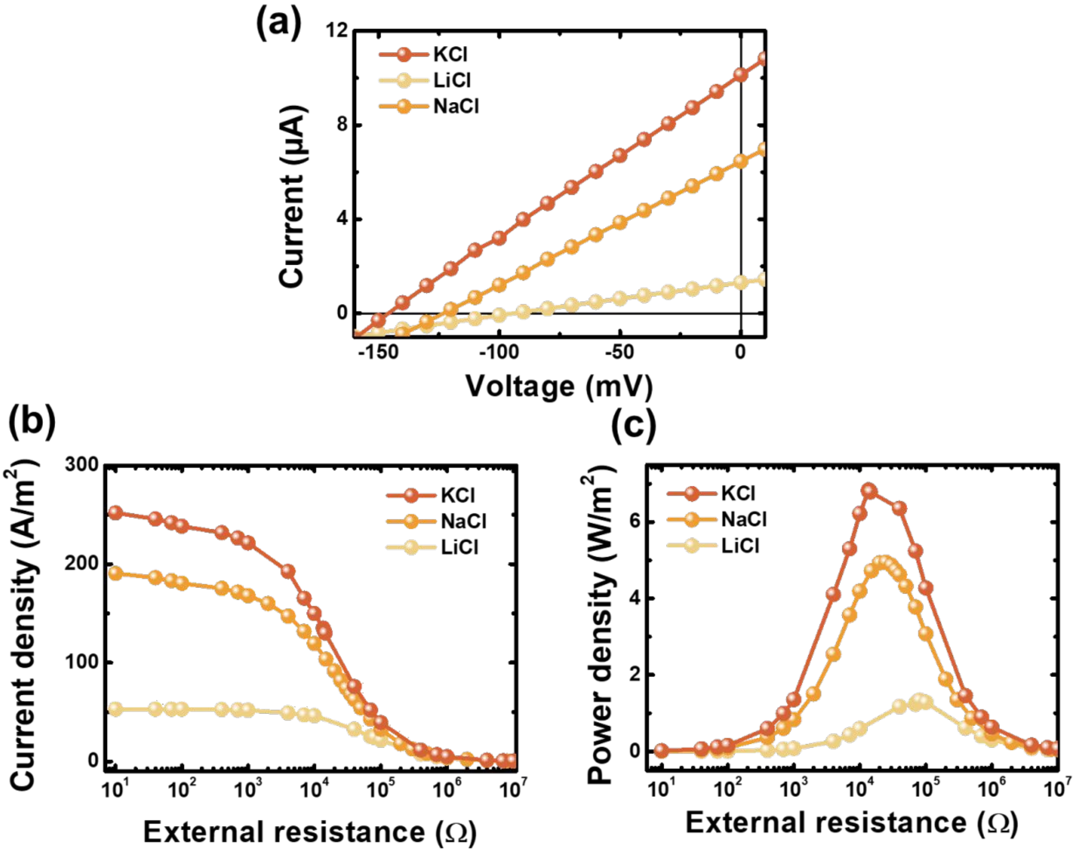

Figure S12. Illustrated (a) *I-V* responses, (b) current densities, and (c) power densities of PAMPS@ANM in different electrolyte solutions at a 50-fold concentration gradient. The maximum power densities with KCl, NaCl and LiCl were 6.84, 4.94, and 1.33 W/m^2, respectively.

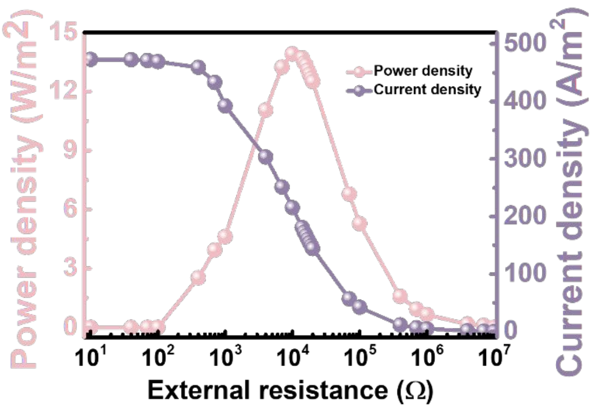

Figure S13. Illustrated current and power densities of PAMPS@ANM under a 50-fold RbCl concentration gradient. The maximum power density achieved was about 13.9 W/m^2.

Table S3. Hydrated radii and diffusion coefficients of the four monovalent alkali cations considered in the present work. P_{max} is the achieved maximum power density measured under a 500 mM/10 mM concentration gradient of using the corresponding monovalent chloride salt.

Cation M^+ in chloride salt	Hydrated radius[13] (Å)	Diffusion coefficient[14] (10^{-9} m^2s^{-1})	P_{max} (W/m^2)
Li^+	3.82	1.03	1.33
Na^+	3.58	1.33	4.94
K^+	3.31	1.96	6.84
Rb^+	3.29	2.07	13.9

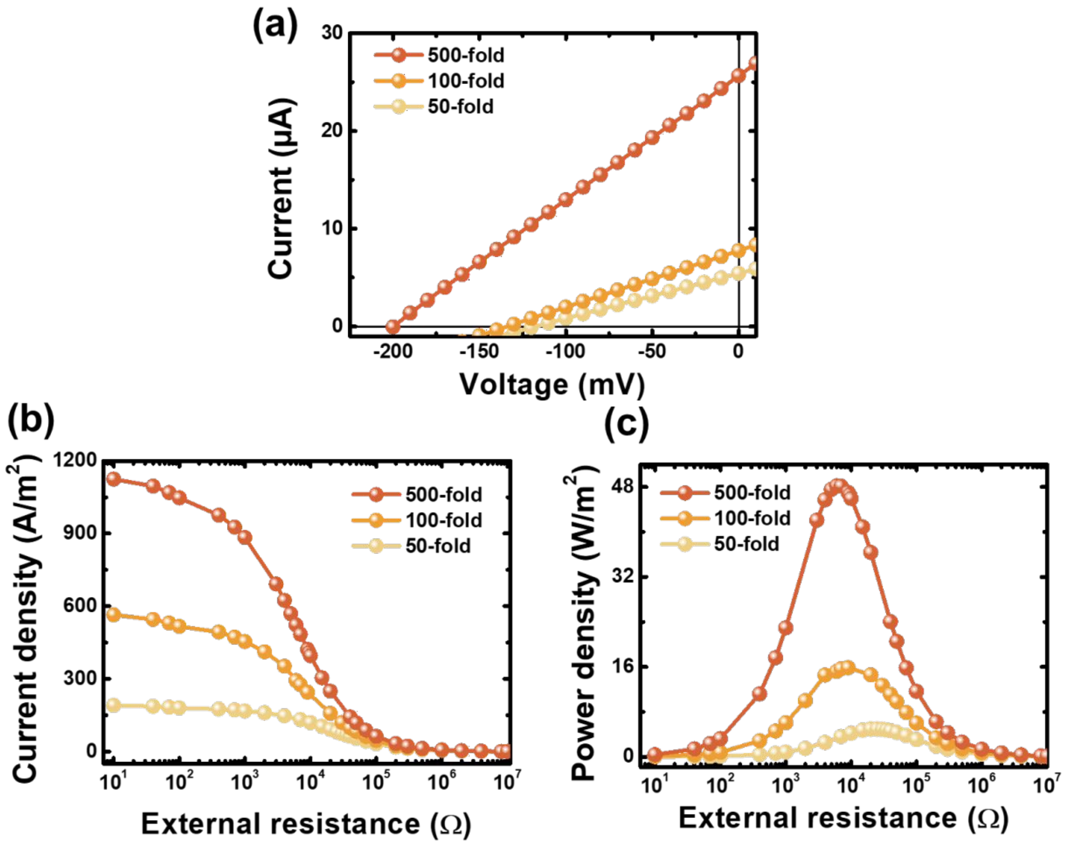

Figure S14. Illustrated (a) *I-V* responses, (b) current densities, and (c) power densities of PAMPS@ANM under different NaCl concentration gradients. The dilute concentration was fixed at 10 mM. The maximum power densities at 50-, 100-, and 500-fold NaCl gradients were 4.94, 15.9, and 48.2 W/m^2, respectively.

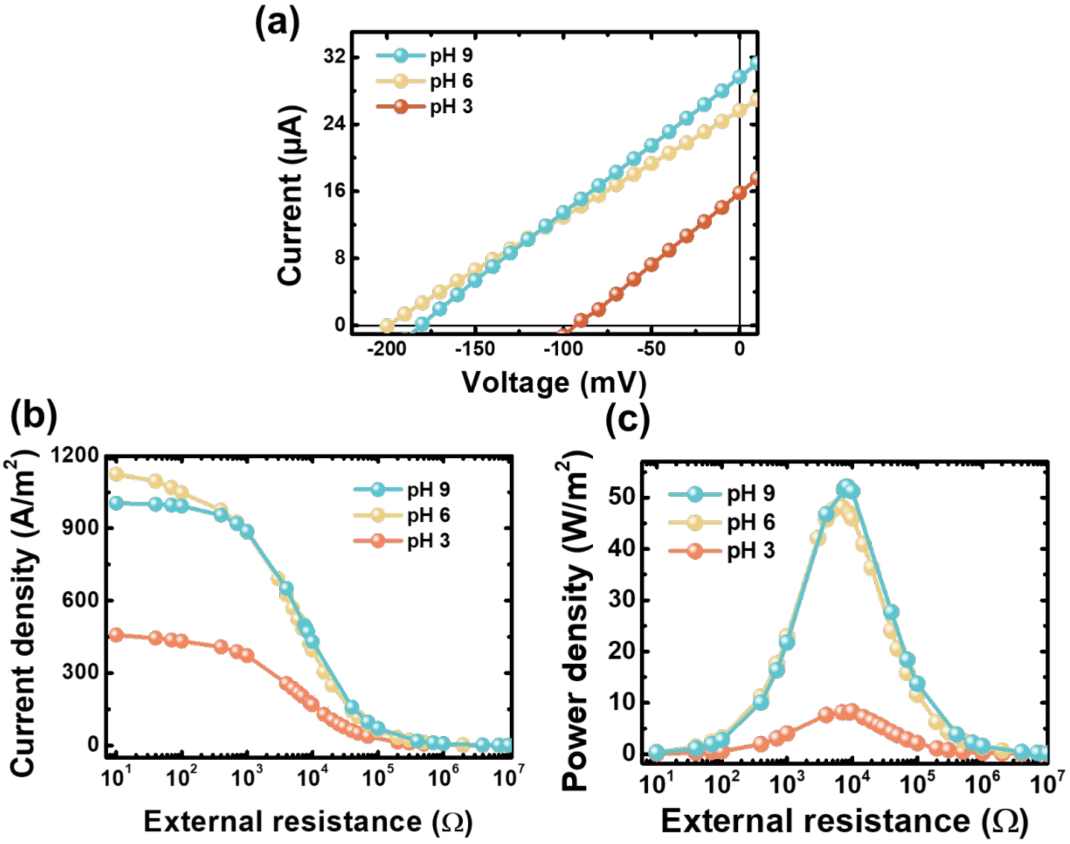

Figure S15. Illustrated (a) *I-V* responses, (b) current densities, and (c) power densities of PAMPS@ANM under a 500-fold NaCl concentration gradient with different pH levels. The maximum power densities achieved under pH 3, pH 6, and pH 9 were 8.32, 48.2, and 52.1 W/m^2, respectively.

Table S4. Comparisons of the practical power density produced from the present nanoconfined hydrogels (PAMPS@ANM) with that from the ion-selective membranes without[15-22] and with[23-29] the ionic diode effect under a 5 M/0.01 M NaCl gradient at room temperature with the same testing area of 0.03 mm^2.

Ion-selective membrane	Resistance (kΩ)	P_{max} (W/m^2)	Reference
GO/CNF	18	13.25	[15]
SPEEK-MOF/SPSF	15	24	[16]
GO/SNF/GO	20	16.2	[17]
bsGOM	25	18.8	[18]
MoS_2/CNF	10.8	15.6	[19]
PAAc@PC	11	11.72	[20]
KANF	8	15	[21]
Cu-TCPP@SNF	6.5	29.5	[22]
CS/SA	8	19.41	[23]
SPEEK/AAO/PPy	1.8	26.22	[24]
SSGOM	10	24.86	[25]
Nafion/ANM	9.8	22.1	[26]
TFP-TPA COF@ANM	3	27.8	[27]
ZGDHM	5.2	49.6	[28]
MXene/ZIF-8	1.2	48.05	[29]
PAMPS@ANM	6	48.2 (pH 6)	This work
PAMPS@ANM	8	52.1 (pH 9)	This work

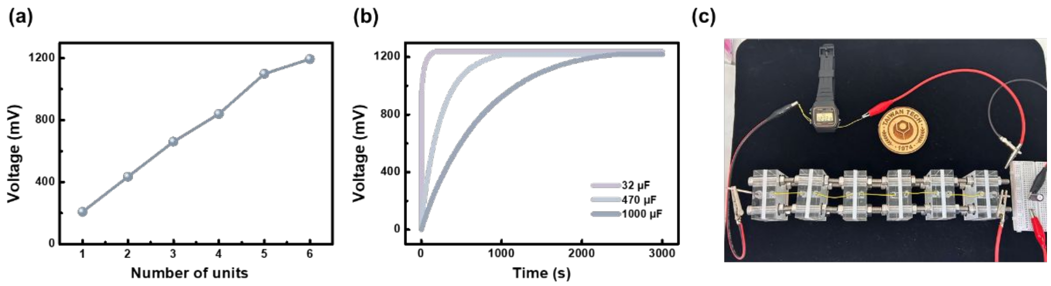

Figure S16. Demonstration of practical applications by the developed nanoconfined hydrogels. (a) The output voltage is proportional to the number of units of the proposed osmotic energy generators connecting in series. (b) The series circuit device built with six units of generators works for charging commercial supercapacitors with a variety of capacitances (32, 470, and 1000 μF) to ~1.2 V within 5 min. (c) Photo illustrating that a series circuit device for powering a digital watch and charging supercapacitors.

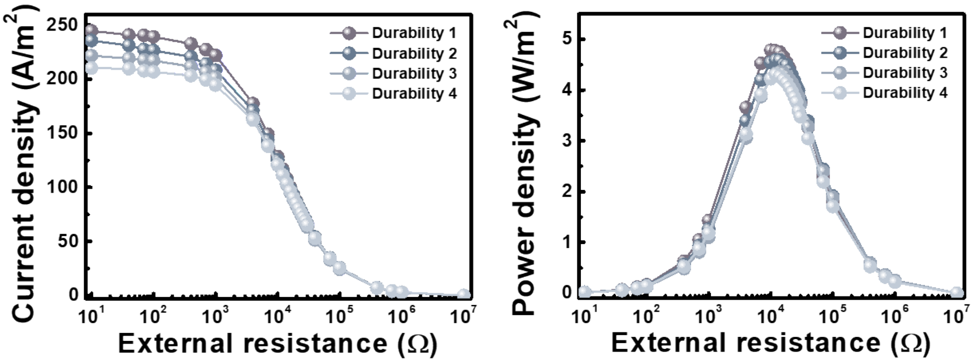

Figure S17. Durability tests of the osmotic energy harvesting performance of PAMPS@ANM in one day, with the achieved maximum power densities of 4.78, 4.59, 4.35, and 4.30 W/m^2. The used concentration gradient was 500 mM/10 mM NaCl.

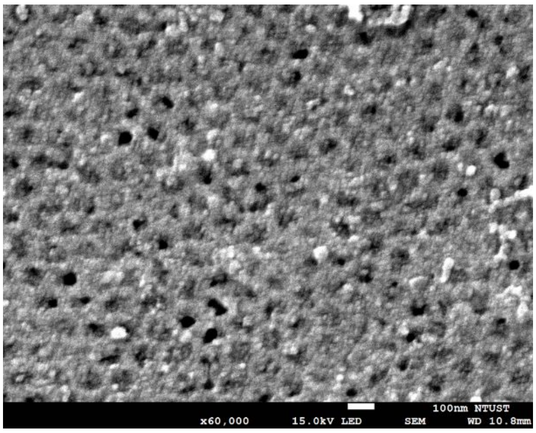

Figure S18. SEM image of PAMPS@ANM captured after the durability test, showing the similar porous structure as the pristine one revealed in Figure 1b.

References

(1) Liu, Y. C.; Yeh, L. H.; Zheng, M. J.; Wu, K. C. W., Highly Selective and High-Performance Osmotic Power Generators in Subnanochannel Membranes Enabled by Metal-Organic Frameworks. *Sci. Adv.* **2021**, *7*, eabe9924.

(2) Fauziah, A. R.; Chu, C. W.; Yeh, L. H., Engineered Subnanochannel Ionic Diode Membranes Based on Metal-Organic Frameworks for Boosted Lithium Ion Transport and Osmotic Energy Conversion in Organic Solution. *Chem. Eng. J.* **2023**, *452*, 139244.

(3) Su, Y. S.; Hsu, S. C.; Peng, P. H.; Yang, J. Y.; Gao, M. Y.; Yeh, L. H., Unraveling the Anomalous Channel-Length-Dependent Blue Energy Conversion Using Engineered Alumina Nanochannels. *Nano Energy* **2021**, *84*, 105930.

(4) Fauziah, A. R.; Yeh, L. H., Engineered Heterogenous Subnanochannel Membranes with a Tri-Continuous Pore Structure of Large Geometry Gradient for Massively Enhanced Osmotic Power Conversion from Organic Solutions. *Adv. Funct. Mater.* **2024**, *34*, 2306834.

(5) Liu, C. T.; Lin, Y. L.; Chu, C. W.; Chang, C. W.; Chiu, Y. J.; Chiu, T. Y.; Lee, L. R.; Chen, J. T., Asymmetries in Porous Membranes: Fabrication of Anodic Aluminum Oxide Membranes with Double-Sized Nanopores and Controlled Surface Properties. *J. Phys. Chem. C* **2019**, *123*, 14540-14546.

(6) Xin, W.; Zhang, Z.; Huang, X.; Hu, Y.; Zhou, T.; Zhu, C.; Kong, X.-Y.; Jiang, L.; Wen, L., High-performance silk-based hybrid membranes employed for osmotic energy conversion. *Nat. Commun.* **2019**, *10*, 3876.

(7) Zhu, X.; Hao, J.; Bao, B.; Zhou, Y.; Zhang, H.; Pang, J.; Jiang, Z.; Jiang, L., Unique ion rectification in hypersaline environment: A high-performance and sustainable power generator system. *Sci. Adv.* **2018**, *4*, eaau1665.

(8) Liu, Y.-C.; Yeh, L.-H.; Zheng, M.-J.; Wu, K. C.-W., Highly selective and high-performance osmotic power generators in subnanochannel membranes enabled by metal-organic frameworks. *Sci. Adv.* **2021**, *7*, eabe9924.

(9) Zhang, Z.; Kong, X. Y.; Xiao, K.; Xie, G. H.; Liu, Q.; Tian, Y.; Zhang, H. C.; Ma, J.; Wen, L. P.; Jiang, L., A Bioinspired Multifunctional Heterogeneous Membrane with Ultrahigh Ionic Rectification and Highly Efficient Selective Ionic Gating. *Adv. Mater.* **2016**, *28*, 144-150.

(10) Zeng, Z.; Ai, Y.; Qian, S., pH-Regulated Ionic Current Rectification in Conical Nanopores Functionalized with Polyelectrolyte brushes. *Phys. Chem. Chem. Phys.* **2014**, *16*, 2465-2474.

(11) Lin, C. Y.; Combs, C.; Su, Y. S.; Yeh, L. H.; Siwy, Z. S., Rectification of Concentration Polarization in Mesopores Leads To High Conductance Ionic Diodes and High Performance Osmotic Power. *J. Am. Chem. Soc.* **2019**, *141*, 3691-3698.

(12) Tsai, P. C.; Su, Y. S.; Gao, M.; Yeh, L. H., Realization of Robust Mesoscale Ionic Diodes for Ultrahigh Osmotic Energy Generation at Mild Neutral pH. *J. Mater. Chem. A* **2021**, *9*, 20502-20509.

(13) Nightingale, E. R., Phenomenological Theory of Ion Solvation. Effective Radii of Hydrated Ions. *J. Phys. Chem.* **1959**, *63*, 1381-1387.

(14) Vanýsek, P., Ionic Conductivity and Diffusion at Infinite Dilution. In *Handbook of Chemistry and Physics*, CRC Press: Boca Raton, 1992; pp (5-111)-(5-113).

(15) Wu, Y. D.; Xin, W. W.; Kong, X. Y.; Chen, J. J.; Qian, Y. C.; Sun, Y.; Zhao, X. L.; Chen, W. P.; Jiang, L.; Wen, L. P., Enhanced Ion Transport by Graphene Oxide/Cellulose Nanofibers Assembled Membranes for High-performance Osmotic Energy Harvesting. *Mater. Horiz.* **2020**, *7*, 2702-2709.

(16) Zhao, X. L.; Lu, C. X.; Yang, L. S.; Chen, W. P.; Xin, W. W.; Kong, X. Y.; Fu, Q.; Wen, L. P.; Qiao, G.; Jiang, L., Metal Organic Framework Enhanced SPEEK/SPSF Heterogeneous Membrane for Ion Transport and Energy Conversion. *Nano Energy* **2021**, *81*, 105657.
(17) Xin, W. W.; Xiao, H. Y.; Kong, X. Y.; Chen, J. J.; Yang, L. S.; Niu, B.; Qian, Y. C.; Teng, Y. F.; Jiang, L.; Wen, L. P., Biomimetic Nacre-Like Silk-Crosslinked Membranes for Osmotic Energy Harvesting. *ACS Nano* **2020**, *14*, 9701-9710.
(18) Qian, Y. J.; Liu, D.; Yang, G. L.; Wang, L. F.; Liu, Y. C.; Chen, C.; Wang, X. A.; Lei, W. W., Boosting Osmotic Energy Conversion of Graphene Oxide Membranes via Self-Exfoliation Behavior in Nano-Confinement Spaces. *J. Am. Chem. Soc.* **2022**, *144*, 13764-13772.
(19) Zhu, C. C.; Liu, P.; Niu, B.; Liu, Y. N.; Xin, W. W.; Chen, W. P.; Kong, X. Y.; Zhang, Z.; Jiang, L.; Wen, L. P., Metallic Two-Dimensional MoS2 Composites as High-Performance Osmotic Energy Conversion Membranes. *J. Am. Chem. Soc.* **2021**, *143*, 1932-1940.
(20) Chen, W. P.; Zhang, Q. R.; Qian, Y. C.; Xin, W. W.; Hao, D. Z.; Zhao, X. L.; Zhu, C. C.; Kong, X. Y.; Lu, B. Z.; Jiang, L.; Wen, L. P., Improved Ion Transport in Hydrogel-Based Nanofluidics for Osmotic Energy Conversion. *ACS Central Sci.* **2020**, *6*, 2097-2104.
(21) Ding, L.; Xiao, D.; Zhao, Z. H.; Wei, Y. Y.; Xue, J.; Wang, H. H., Ultrathin and Ultrastrong Kevlar Aramid Nanofiber Membranes for Highly Stable Osmotic Energy Conversion. *Adv. Sci.* **2022**, *9*, 2202869.
(22) Mai, V. P.; Gu, C. R.; Fauziah, A. R.; Yang, Z. J.; Wu, K. C. W.; Yeh, L. H.; Yang, R. J., Two-Dimensional Metal-Organic Framework Nanocomposite Membranes with Shortened Ion Pathways for Enhanced Salinity Gradient Power Harvesting. *Chem. Eng. J.* **2024**, *484*, 149649.
(23) Bian, G. S.; Pan, N.; Luan, Z. H.; Sui, X.; Fan, W. X.; Xia, Y. Z.; Sui, K. Y.; Jiang, L., Anti-Swelling Gradient Polyelectrolyte Hydrogel Membranes as High-Performance Osmotic Energy Generators. *Angew. Chem.-Int. Edit.* **2021**, *60*, 20294-20300.
(24) Hao, J. R.; Bao, B.; Zhou, J. J.; Cui, Y. S.; Chen, X. C.; Zhou, J. L.; Zhou, Y. H.; Jiang, L., A Euryhaline-Fish-Inspired Salinity Self-Adaptive Nanofluidic Diode Leads to High-Performance Blue Energy Harvesters. *Adv. Mater.* **2022**, *34*, 2203109.
(25) Liu, S. H.; Zhang, D.; Fang, Y. P.; Liang, Z. X.; Zhao, Z. K.; Chen, X. C.; Yao, J. M.; Jiang, L., Topologically Programmed Graphene Oxide Membranes with Bioinspired Superstructures toward Boosting Osmotic Energy Harvesting. *Adv. Funct. Mater.* **2023**, *33*, 2211532.
(26) Chang, C. W.; Chu, C. W.; Su, Y. S.; Yeh, L. H., Space Charge Enhanced Ion Transport in Heterogeneous Polyelectrolyte/Alumina Nanochannel Membranes for High-Performance Osmotic Energy Conversion. *J. Mater. Chem. A* **2022**, *10*, 2867-2875.
(27) Gao, M. Y.; Zheng, M. J.; El-Mahdy, A. F. M.; Chang, C. W.; Su, Y. C.; Hung, W. H.; Kuo, S. W.; Yeh, L. H., A Bioinspired Ionic Diode Membrane Based on Sub-2 nm Covalent Organic Framework Channels for Ultrahigh Osmotic Energy Generation. *Nano Energy* **2023**, *105*, 108007.
(28) Huang, K. T.; Hung, W. H.; Su, Y. C.; Tang, Y. C.; Linh, L. D.; Huang, C. J.; Yeh, L. H., Zwitterionic Gradient Double-Network Hydrogel Membranes with Superior Biofouling Resistance for Sustainable Osmotic Energy Harvesting. *Adv. Funct. Mater.* **2023**, *33*, 2211316.

(29) Zhou, J.; Hao, J.; Wu, R.; Su, L.; Wang, J.; Qiu, M.; Bao, B.; Ning, C.; Teng, C.; Zhou, Y.; Jiang, L., Maximizing Ion Permselectivity in MXene/MOF Nanofluidic Membranes for High-Efficient Blue Energy Generation. *Adv. Funct. Mater.* **2022**, *32*, 2209767.
